# Tuning your writing

**DOI:** 10.1007/s40037-017-0346-0

**Published:** 2017-04-10

**Authors:** Christopher Watling

**Affiliations:** Office of Postgraduate Medical Education, Schulich School of Medicine and Dentistry, Western University, London, Ontario Canada

In the Writer’s Craft section we offer simple tips to improve your writing in one of three areas: Energy, Clarity and Persuasiveness. Each entry focuses on a key writing feature or strategy, illustrates how it commonly goes wrong, teaches the grammatical underpinnings necessary to understand it and offers suggestions to wield it effectively. We encourage readers to share comments on or suggestions for this section on Twitter, using the hashtag: #how’syourwriting?

Perhaps you have winced, on occasion, while re-reading something you have written – something that just doesn’t *sound *right. As an avid reader of *The Writer’s Craft, *you have considered the usual suspects – clumsy sentence construction, faulty grammar, unnecessary words – but they are all behaving themselves. The problem may lie in two more elusive elements of writing: tone and voice. These qualities impact how readers think and feel about your subject matter – and about you. Gaining control of tone and voice will enhance your versatility as a writer, enable you to more effectively join – or lead – conversations, and allow you to provoke, challenge, or inspire your readers.

## Tone

Tone reflects your stance toward your subject [1]. Your tone can range from devoted to dismissive, from collaborative to confrontational. We often assume that scientific writing demands a neutral tone, as if the writer has no relationship to the subject matter. But the best scientific writers skilfully modulate tone to craft more powerful research stories.

Tone pervades a paper’s introduction and literature review, conveyed by the words and phrases used to map gaps in the existing literature and to carve a place for the study to be described. Verbs set the tone; used carelessly, they can send the wrong message. Consider the following:

*Researchers exploring how individuals respond to feedback have consistently* ***failed*** *to account for the influence of context.*

versus:

*Researchers have* ***advanced*** *our understanding of how individuals respond to feedback; we now must* ***explore*** *how context shapes this dynamic.*

In the first sentence, the tone is judgmental, the verb ‘failed’ serving to criticize existing research as deficient. In the second sentence, the tone is diplomatic, acknowledging that others ‘have advanced’ the knowledge, while still making the case that context deserves exploration. The focus on what the author plans to add, rather than on what others have failed to do, builds a tone of collaboration rather than antagonism.

Both approaches are defensible, provided they are purposeful. Sometimes a scrappy, critical tone is your aim as a writer, particularly if you are a veteran of a particular research conversation. Referencing my colleague Lorelei Lingard’s work, I could write something like this:

*Lingard’s exquisite prose deflects attention from the key issue: doctors simply don’t consider other health professionals as equal members of the team.*

Here, I establish a brash tone with the suggestion that Lingard has used her impeccable writing skills to befuddle readers, and I reinforce that tone with a boldly worded declaration of the ‘real’ issue. As a senior scholar and colleague, I might get away with this kind of verbal sparring. But if I were a newcomer to this conversation, I might opt instead for a respectful, collegial tone:

*Lingard’s work sheds valuable light on team competence, but the influence of power and hierarchy requires further attention.*

This version acknowledges Lingard’s contribution to the conversation without judgment, then prudently introduces a new conversational thread.

Discussion sections, too, brim with tone. Here, the high-wire act involves balancing confidence in the novelty and originality of your work with respect for the work of others in your domain. Overconfident prose can read as arrogant – or worse, as naïve. Verbs, again, can work for us or against us (see Table 1).

**Table 1:** Choosing verbs

Verbs that may over-reach …	Judicious alternatives
We have established …	We speculate …
Our work proves …	Our work suggests …
We demand …	We propose …

Adverbs may also contribute to a tone that condescends to readers; words like ‘clearly’, ‘obviously’, and ‘interestingly’ are best avoided. Where adverbs are concerned, often the most judicious alternative is no adverb at all.

A cautious tone need not dilute the message. You can be clear about your contributions without arrogance. For example:

*Our work builds on existing research on workplace learning, and we add two new insights that may influence future exploration in this arena.*

Here, the ‘so what’ message is presented matter-of-factly, while acknowledging how it integrates with an existing conversation.

## Voice

In a previous *Writer’s Craft*, the active and the passive voices were distinguished in relation to verb use [2]. Here, I use the term ‘voice’ differently, referring instead to the *writer’s voice* and how it comes through in their written work. Voice ‘creates the illusion that the writer is speaking directly to the reader from the page ’ [3].

For poets, novelists, journalists, songwriters, playwrights, and screenwriters, voice is everything – their success depends on establishing a voice that is distinct and recognizable. For academic writers, establishing a distinctive voice can be challenging. For one thing, authorship is typically shared; writing as ‘we’ rather than as ‘I’ may stifle an individual’s voice. For another, the genres in which we write can confine us, seeming to leave little room for unique voices. But if you think of the academic writers whose work you most admire, you can likely find in their words something individual and original.

Even within the constraints of the research paper genre, you can make your voice heard. Your voice will emerge most naturally when you write with a goal of engaging your reader. Don’t be afraid, even in a research paper, to choose verbal and grammatical strategies that captivate and persuade, particularly at key moments in your argument. Mix up sentence length and structure, choosing simple sentences to emphasize key points [3]. Consider the following:

*While validity is undoubtedly important in assessment, reliability must also be taken into account; when the stakes of an assessment event are high, both qualities deserve careful consideration.*

*Validity and reliability share the assessment stage. For high-stakes assessment, both matter.*

In the first example, the idea is expressed through a compound-complex sentence. In the second, two simple sentences are used instead, along with a metaphor (sharing the stage). Both are correct; what differs is voice. The first approach is more typical of academic writing, but the second is arguably more conversational and engaging. What’s more, the reader is more apt to remember the key message.

While the research paper format affords limited opportunities for voice to emerge, other genres offer more flexibility for researchers, educators, and academics. Commentaries, opinion pieces, letters to the editor, blogs, methodological guides – these genres provide great opportunities to play with voice, as they are less rule-bound. In these settings, writers often aim for a conversational voice, and several strategies can help. The use of first- or second-person pronouns fosters the sense that the author is speaking directly to the reader [4]:

*When I write in the first person, it connects me more readily to my reader *(first person).

*You can develop your own voice by experimenting with word choice and sentence length *(second person).

Both these sentences reach out directly to the reader. Rendered in third person, the sentence is less captivating:

*When a writer uses first person, it connects them more readily to their readers.*

The use of conversational idioms over formal, academic language creates the feeling of a discussion between writer and reader. Contractions, for example, may be copy-edited out of research papers, but can usefully establish a conversational voice in other settings. For example:

*Don’t worry about the stylistic details when writing a first draft.*

Here, ‘don’t’ works much more convincingly that ‘do not’; the latter would create an undesirable distance between writer and reader.

Experiments with evocative metaphors further hone voice. Rachel Ellaway, writing about how music has influenced her as a scholar, wrote:

*I abandoned piano lessons early on, my door to musicianship seemingly slammed shut by tired practice pieces and regular reminders of my myriad musical failings *[5].

The words jump off the page, not only evoking the story, but also creating the illusion that Rachel is telling the story directly to you. She accomplishes this feat by writing in first person, and by using the metaphor of a door slamming shut to colourfully express her early frustrations with music lessons. So follow her lead, grabbing opportunities to write in genres outside your typical academic scope; the voice you develop there may seep into even your most traditionally academic writing, and your work will be stronger for it.

## Getting it right

Tone and voice are tricky. Read your work out loud – if you wince, consider whether to modulate tone or voice for better effect. Ask a trusted colleague to read your work-in-progress, and ask: Have I struck the right chords? How does the writing make you feel? How would a reviewer react to this sentence or that? Does it sound like *me*? Aspire to engage readers, leaving them feeling they’ve just had a great conversation.
